# Consumer perceptions of legal cannabis products in Canada, 2019–2021: a repeat cross-sectional study

**DOI:** 10.1186/s12889-022-14492-z

**Published:** 2022-11-08

**Authors:** Elle Wadsworth, Fathima Fataar, Samantha Goodman, Danielle M. Smith, Justine Renard, Robert Gabrys, Rebecca Jesseman, David Hammond

**Affiliations:** 1 School of Public Health Sciences, University of Waterloo, 200 University Ave W, Waterloo, ON N2L 3G1 Canada; 2 Canadian Centre On Substance Use and Addiction, 75 Albert St, Suite 500, Ottawa, ON K1P 5E7 Canada; 3 Department of Health Behavior, Roswell Park Comprehensive Cancer Center, Elm and Carlton Streets, Buffalo, NY 14263 USA; 4 Mental Health and Addictions, Health PEI, 115 Deacon Grove Land, Charlottetown, PE C1A 7N5 Canada

**Keywords:** Cannabis, Marijuana, Legalization, Perceptions, Price, Safety

## Abstract

**Background:**

Consumer perceptions of legal cannabis products may drive willingness to purchase from the illegal or legal market; however, little is known on this topic. The current study examined perceptions of legal products among Canadian cannabis consumers over a 3-year period following federal legalization of non-medical cannabis in 2018.

**Methods:**

Data were analyzed from Canadian respondents in the International Cannabis Policy Study, a repeat cross-sectional survey conducted in 2019–2021. Respondents were 15,311 past 12-month cannabis consumers of legal age to purchase cannabis. Weighted logistic regression models examined the association between perceptions of legal cannabis and province of residence, and frequency of cannabis use over time.

**Results:**

In 2021, cannabis consumers perceived legal cannabis to be safer to buy (54.0%), more convenient to buy (47.8%), more expensive (47.2%), safer to use (46.8%) and higher quality (29.3%) than illegal cannabis. Except for safety of purchasing, consumers had more favourable perceptions of legal cannabis in 2021 than 2019 across all outcomes. For example, consumers had higher odds of perceiving legal cannabis as more convenient to buy in 2021 than 2019 (AOR = 3.09, 95%CI: 2.65,3.60). More frequent consumers had less favourable perceptions of legal cannabis than less frequent consumers.

**Conclusions:**

Three years since legalization, Canadian cannabis consumers generally had increasingly favourable perceptions of legal vs. illegal products – except for price – with variation across the provinces and frequency of cannabis use. To achieve public health objectives of legalization, federal and provincial governments must ensure that legal cannabis products are preferred to illegal, without appealing to non-consumers.

**Supplementary Information:**

The online version contains supplementary material available at 10.1186/s12889-022-14492-z.

## Background

Canada legalized non-medical cannabis in October 2018. One of the primary objectives of the *Cannabis Act* is to remove the illegal market and provide a regulated cannabis market for consumers [1]. In order to transition consumers to the legal market, there must be incentive to do so, whether that be removal of criminal risk, cheaper price, or higher quality. Understanding how legal products are perceived by consumers, and whether perceptions change over time, is important for policymakers and public health researchers seeking to understand the impacts of legalization, and to maximize public health and safety benefits.

Previous research demonstrates that there has been a decline in the reported purchase of illegal cannabis since legalization, and subsequent increase in the reported purchase of legal cannabis [2–8]. Data from the Canadian Cannabis Survey—the Canadian government’s annual national cannabis survey – showed that 53% of past 12-month cannabis consumers reported ‘usually’ sourcing cannabis from a legal store, an increase from 41% in 2020 and 29% in 2019 [2–4]. Regardless of increasing interaction with the legal market, there is still a substantial proportion of consumers ‘usually’ sourcing illegally three years post-legalization [2–4].

The legal cannabis market in Canada has expanded and evolved in the three years since legalization, and the extent has varied across the provinces. First, not all cannabis products entered the legal market in October 2018. Dried flower and some orally administered oils were available for purchase beginning in October 2018. Edibles, topicals, and extracts were available in legal stores from December 2019 [9]. Québec—the most populated province with a government-run retail market—was the only province to impose further restrictions on legal cannabis products: all products are restricted to a maximum tetrahydrocannabinol (THC) content of 30%, and edibles cannot be attractive to consumers under 21 years of age, including prohibition of cannabis-infused candy, chocolate, or confections [10]. Second, while the average price of legal dried flower has decreased since legalization [11] and is increasingly aligning with the price of illegal dried flower [8, 12], legal cannabis has remained more expensive than illegal cannabis since legalization. This presents a challenge for governments which must strike a balance whereby the price is low enough to encourage consumers to transition from the illegal market, but not low enough to encourage greater consumption or initiation. Frequent cannabis consumption has been linked to various negative health effects such as the cardiovascular effects of smoking cannabis, risk of dependence, mental health conditions, as well as negative consequences among young people such as poor academic achievement [13–22]. Third, the number of physical retail stores in Canada has increased fourfold since legalization, from 508 in September 2019, to 2465 in September 2021 [23]. Of note, this increase in retail stores has not been evenly distributed across the provinces due to between-province differences in the overall establishment of retail markets, as well as within-province changes in the number of retail stores permitted. For instance, Ontario saw the majority of new stores open several years after legalization: from 24 stores in 2019 to 1042 stores in 2021, whereas New Brunswick and Prince Edward Island have retained the same number of stores since legalization [24, 25]. Québec, the second largest province in Canada, had the lowest stores per capita in 2020 and 2021. It is unknown how these key changes in products, price, and retail stores have influenced perceptions of the legal market across the provinces.

Few studies have explored the perceptions of cannabis products from legal markets. The Canadian Cannabis Survey documents self-reported factors that provide the greatest influence on where consumers source their cannabis [2–4]. In 2019–2021, the top four factors were price, obtaining cannabis safely, quality, and convenience. Indeed, research examining reasons for purchasing in the Canadian illegal market concluded the most common reasons to be price, convenience, quality, and loyalty to dealers [26]. In a U.S. study examining perceptions of legal cannabis products in 2018 among states that had legalized non-medical cannabis, consumers had favourable perceptions of legal cannabis on most factors with the exception of price, which they viewed as more expensive than illegal cannabis [27]. In the same U.S. study, perceptions of legal products varied by cannabis use, where frequent cannabis consumers (vs. never consumers) had more favourable perceptions of legal cannabis products [27]. There is little research to date on whether perceptions of legal cannabis in Canada vary over time, by province, or by frequency of cannabis use.

Research on the perceptions of cannabis products in legal markets is important to understand so that governments can enact provisions which persuade existing consumers to transition away from the illegal market after legalization and fully achieve the public health and safety objectives of legalization. With retail policies varying across the provinces, there are effectively 10 legal markets, one for each province. It is important that each provincial government understands how legal cannabis is perceived so they can maximize transition into their legal market. Studying perceptions across provinces also provides the opportunity to explore possible relationships between retail approaches and consumer perceptions. The aims of this study were to examine: 1) the perceptions of legal cannabis products three years post-legalization among past 12-month cannabis consumers of provincial legal age to purchase cannabis; and 2) the association between perceptions of legal cannabis products, province, and frequency of cannabis use in 2019–2021.

## Methods

Data are from Waves 2–4 of the International Cannabis Policy Study (ICPS), which consists of repeat cross-sectional surveys conducted in Canada and the U.S. Data were collected via self-completed web-based surveys in September–October in 2019, 2020, and 2021 from respondents aged 16–65. A non-probability sample of respondents was recruited through the Nielsen Consumer Insights Global Panel and their partners’ panels. For the ICPS surveys, Nielsen draws stratified random samples from the online panels, with quotas based on age and state/province of residence. Nielsen emailed panelists an invitation to access the ICPS survey via a unique link; respondents are unaware of the survey topic prior to accessing the link. Respondents confirmed their eligibility and provided consent before completing the survey. Surveys were conducted in English or French. Median survey time was 25 min in 2019, 21 min in 2020, and 22 min in 2021. Upon completion, respondents receive remuneration in accordance with their panel’s usual incentive structure.

The study was reviewed by and received ethics clearance through a University of Waterloo Research Ethics Committee (ORE#31,330). A full description of the study methods can be found in the ICPS Technical Reports and methodology paper [28–31].

### Measures

#### Socio-demographic measures

Gender, age, ethnicity/race, highest education level, perceived income adequacy, device type used to complete survey, and province of residence. For gender (Cis man/Trans man/Cis woman/Trans woman/Unstated), those who answered “Other” were excluded due to very small cell sizes (n_2019_ = 23; n_2020_ = 21; n_2021_ = 1). In regression models, gender was further categorized to: “Man”, “Woman”, “Unstated”. Gender and sex at birth were both collected separately in the survey; however, gender was chosen due to questions focusing on perceptions of products rather than issues related to biological factors [32]. Minimum legal age to purchase cannabis (MLA) was taken from provincial laws in September 2019, 2020, and 2021. For “perceived income adequacy” and “highest level of education”, those who answered, “Don’t know” or “Refuse to answer” were categorized to “Not stated”. See Table 1 for response options.

**Table 1:** Sample characteristics of past 12-month cannabis consumers of legal age to purchase cannabis in Canada in 2019–2021

	**Unweighted % (n)**			**Weighted % (n)**		
	**2019** *n* = 4,857	**2020** *n* = 4,652	**2021** *n* = 5,802	**2019** *n* = 4,867	**2020** *n* = 4,728	**2021** *n* = 5,716
**Age group**						
MLA-25	14.5 (702)	11.6 (540)	10.1 (584)	13.8 (672)	9.5 (450)	12.8 (732)
26–35	26.6 (1293)	24.4 (1133)	26.2 (1521)	30.5 (1482)	31.3 (1480)	29.9 (1707)
36–45	23.1 (1121)	22.2 (1033)	25.0 (1448)	22.5 (1094)	23.7 (1120)	24.9 (1422)
46–55	18.0 (876)	19.9 (921)	17.3 (1002)	18.4 (894)	19.0 (896)	17.2 (985)
56–65	17.8 (865)	22.0 (1025)	21.5 (1247)	14.9 (726)	16.5 (782)	15.2 (870)
**Gender**						
Woman	57.6 (2785)	60.2 (2790)	56.6 (3285)	44.9 (2175)	46.6 (2196)	46.5 (2656)
Cis woman	*57.2 (2765)*	*59.8 (2769)*	*56.3 (3267)*	*44.2 (2143)*	*46.0 (2170)*	*46.0 (2628)*
Trans woman	*0.4 (20)*	*0.5 (21)*	*0.3 (18)*	*0.6 (31)*	*0.6 (26)*	*0.5 (28)*
Man	41.3 (1997)	38.7 (1793)	42.5 (2462)	53.7 (2603)	51.6 (2434)	52.2 (2985)
Cis man	*40.6 (1964)*	*38.3 (1775)*	*42.1 (2443)*	*53.2 (2579)*	*51.4 (2423)*	*51.9 (2966)*
Trans man	*0.7 (33)*	*0.4 (18)*	*0.3 (19)*	*0.5 (24)*	*0.2 (11)*	*0.3 (19)*
Unstated	1.1 (52)	1.1 (51)	0.9 (53)	1.5 (71)	1.8 (84)	1.3 (73)
**Race/Ethnicity**						
White	77.2 (3749)	78.4 (3647)	74.2 (4304)	74.6 (3633)	76.0 (3594)	72.6 (4148)
Other/Mixed	6.9 (334)	6.7 (310)	7.2 (420)	7.6 (368)	6.7 (317)	8.0 (458)
East/Southeast Asian	4.6 (222)	4.6 (212)	5.6 (325)	4.7 (227)	4.9 (232)	5.0 (288)
Black	3.1 (150)	2.5 (115)	3.2 (188)	4.0 (193)	3.8 (180)	3.6 (205)
Indigenous	3.9 (187)	3.1 (146)	3.2 (185)	4.0 (193)	2.7 (128)	3.6 (206)
South Asian	2.2 (109)	2.4 (113)	2.8 (163)	2.7 (132)	3.0 (140)	3.1 (176)
Latinx	1.4 (66)	1.2 (56)	2.1 (123)	1.8 (85)	1.6 (76)	2.6 (150)
Middle Eastern	0.8 (40)	1.1 (53)	1.6 (94)	0.7 (36)	1.3 (61)	1.5 (85)
**Education**						
Less than high school	6.2 (303)	6.0 (278)	5.8 (334)	12.1 (587)	9.4 (444)	12.2 (696)
High school diploma	17.4 (844)	16.6 (771)	15.9 (925)	28.0 (1361)	29.4 (1388)	29.0 (1656)
Some college or technical vocation	46.5 (2259)	44.8 (2086)	44.2 (2567)	36.1 (1758)	36.4 (1723)	35.8 (2049)
Bachelor’s degree or higher	28.9 (1404)	31.8 (1480)	33.4 (1935)	22.7 (1106)	24.0 (1133)	22.2 (1270)
Unstated	1.0 (47)	0.8 (37)	0.7 (41)	1.2 (56)	0.9 (41)	0.8 (45)
**Income adequacy**						
Very difficult	11.0 (532)	9.0 (417)	9.8 (566)	10.9 (533)	9.4 (442)	10.8 (615)
Difficult	24.4 (1183)	21.6 (1005)	20.8 (1209)	25.1 (1222)	20.9 (987)	22.0 (126)
Neither easy nor difficult	33.6 (1634)	36.4 (1692)	34.8 (2018)	33.5 (1628)	36.8 (1742)	34.6 (1980)
Easy	19.4 (944)	21.2 (985)	21.0 (1216)	18.6 (906)	21.2 (1003)	19.3 (1101)
Very Easy	9.2 (449)	9.7 (449)	11.5 (668)	8.8 (430)	9.2 (437)	10.3 (590)
Unstated	2.4 (115)	2.2 (104)	2.2 (125)	3.1 (148)	2.5 (118)	3.0 (174)
**Cannabis use frequency**						
Past year, but less than monthly	34.9 (1697)	32.6 (1517)	28.9 (1677)	31.1 (1516)	28.3 (1336)	25.0 (1426)
Monthly	19.2 (933)	18.7 (870)	19.4 (1124)	19.5 (947)	18.8 (890)	19.8 (1130)
Weekly	16.0 (776)	16.2 (755)	17.2 (997)	16.4 (796)	16.7 (789)	16.9 (965)
Daily/almost daily	29.9 (1451)	32.5 (1510)	34.5 (2004)	33.1 (1609)	36.2 (1713)	38.4 (2194)
**Province of residence**						
British Columbia	15.3 (743)	16.8 (782)	14.0 (811)	14.5 (705)	15.1 (713)	15.4 (880)
Alberta	15.6 (757)	15.7 (729)	13.5 (781)	13.0 (633)	12.5 (590)	12.8 (730)
Saskatchewan	5.4 (260)	6.3 (292)	4.8 (279)	3.1 (150)	3.6 (168)	3.0 (174)
Manitoba	6.0 (289)	5.6 (258)	5.3 (308)	3.7 (180)	3.4 (162)	3.8 (220)
Ontario	23.4 (1134)	20.6 (959)	33.3 (1932)	41.1 (1998)	40.6 (1919)	40.6 (2322)
Québec	18.4 (892)	13.5 (626)	12.6 (729)	17.5 (853)	17.6 (830)	17.4 (994)
New Brunswick	5.1 (246)	7.1 (332)	5.6 (322)	2.3 (110)	2.4 (111)	2.3 (129)
Nova Scotia	6.8 (328)	7.1 (328)	6.1 (355)	3.1 (149)	3.0 (141)	2.9 (167)
Prince Edward Island	0.8 (41)	1.3 (62)	1.1 (65)	0.4 (20)	0.4 (19)	0.4 (22)
Newfoundland & Labrador	3.4 (167)	6.1 (284)	3.8 (220)	1.4 (70)	1.6 (75)	1.4 (79)

Income adequacy is assessed by the question: “Thinking about your family’s income, how difficult or easy is it to make ends meet?”, where ‘making ends meet’ means having enough money to pay for the things your family needs

For regression models, Québec was chosen as the reference category for province of residence due to its contrast to most provinces on policy measures, such as MLA, retail structure, and product standards. For example, Québec is the most populated province with a government-run retail structure, has the highest MLA of 21 years, and has restrictions on cannabis products, including a THC limit of 30% across all products [10].

#### Cannabis use frequency

Cannabis use frequency was assessed through questions, “*How often do you use cannabis*?” and “*When was the last time you used cannabis*?” Responses were categorized to: “Less than monthly consumer but used in the past 12 months”, “Monthly consumer”, “Weekly consumer”, and “Daily consumer” (Daily or almost daily consumer).

#### Perception of cannabis from legal sources

Respondents were asked: “*We would like to know how cannabis products from legal/authorized sources compare to cannabis products from illegal/unauthorized sources*” with options 1) “Quality of legal cannabis” (Higher quality/No difference/Lower quality/Don’t know); 2) “Price of legal cannabis” (More expensive/No difference/Less expensive/Don’t know); 3) “Convenience of buying legal cannabis” (More convenient to buy/No difference/Less convenient to buy/Don’t know); 4) “Safety of using legal cannabis” (Safer to use/No difference/Less safe to use/Don’t know); 5) “Safety of buying legal cannabis” (Safer to buy/No difference/Less safe to buy/Don’t know).

All questions included “Don’t know” and “Refuse to answer” options. All “Don’t know’” and “Refuse to answer” options were excluded unless specified within the measures above.

### Analytic sample

The current study reports data from the Canadian ICPS sample only. Among Canadian respondents, the survey had a response rate of 3.7% in 2019, 1.5% in 2020, and 1.8% in 2021. The survey had an American Association for the Public Opinion Research (AAPOR) cooperation rate of 63% in 2019, 66% in 2020, and 68% in 2021 [33]. Respondents were excluded for poor data quality prior to analysis for reasons such as speeding, dishonesty, or duplicate entries (n_2019_ = 1,228; n_2020_ = 1,221; n_2021_ = 1,904) [28–30]. The final Canadian cross-sectional samples comprised of 15,256 respondents in 2019, 15,780 in 2020, and 16,952 in 2021.

Analyses were conducted on the sub-sample of Canadian respondents who had consumed cannabis in the past 12 months and were of provincial legal age to purchase cannabis products (n_2019_ = 4,857; n_2020_ = 4,652; n_2021_ = 5,802). Missing data were removed using case-wise deletion for variables used in regression models: highest level of education (*n* = 125 [0.8%]); quality of legal products (*n* = 101 [0.7%]); price of legal products (*n* = 92 [0.6%]); convenience of buying legal products (*n* = 106 [0.7%]); safety of using legal products (*n* = 103 [0.7%]); and safety of buying legal products (*n* = 95 [0.6%]).

### Statistical analysis

Post-stratification sample weights were constructed based on the Canadian Census estimates. Respondents were classified into age-by-sex-by-province, education, and age-by-tobacco cigarette status groups. A raking algorithm was applied to the cross-sectional analytic samples to compute weights that were calibrated to these groupings. Weights were rescaled to the sample size for all years in Canada. Estimates are weighted unless otherwise specified.

First, descriptive statistics were used to describe the perceptions of legal cannabis products in the past 12 months, 2019–2021. Second, separate multinomial logistic regression models were fitted to examine each of the five perceptions of legal cannabis products and their association between province of residence and cannabis use frequency in 2019–2021 (see measures for categorization). In all models, two-way interactions were conducted for survey wave and cannabis use frequency. All models were adjusted for survey year, province, age, gender, education level, ethnicity/race, income adequacy, and survey device type. Adjusted odds ratios (AORs) are reported with 95% confidence intervals (95% CI). Analyses were conducted using survey procedures in SAS (SAS version 9.4, SAS Institute Inc., Cary, NC, USA).

## Results

Table 1 displays the weighted and unweighted sample characteristics of Canadian past 12-month cannabis consumers of legal age in 2019, 2020, and 2021.

Additional File 1a-e display visual representations of the perceptions of legal cannabis among the study sample, 2019–2021.

### Quality of legal cannabis

Across all years, approximately one-quarter of respondents reported that legal cannabis was of higher quality or no different than illegal cannabis and one-fifth reported legal cannabis was of lower quality (Table 2; Additional File 1a).

**Table 2:** Perceptions of cannabis from legal sources, 2019–2021

	**Cannabis from legal sources vs illegal sources** **% (n)**			
	**2019** *n* = 4,857	**2020** *n* = 4,652	**2021** *n* = 5,802	**Pooled years** *n* = 15,311
**Quality of legal cannabis**				
Higher quality	25.9 (1177)	25.6 (1110)	29.3 (1616)	27.1 (3903)
No difference	24.7 (1159)	26.8 (1145)	27.6 (1445)	26.4 (3749)
Lower quality	19.7 (913)	18.8 (816)	16.3 (895)	18.1 (2624)
Don’t know	29.8 (1568)	28.9 (1558)	26.8 (1808)	28.4 (4934)
**Price of legal cannabis**				
More expensive	57.4 (2763)	50.1 (2348)	47.2 (2721)	51.3 (7832)
No difference	13.9 (623)	17.3 (678)	19.9 (1007)	17.2 (2308)
Less expensive	6.1 (251)	7.8 (313)	9.5 (499)	7.9 (1063)
Don’t know	22.6 (1178)	24.8 (1293)	23.4 (1545)	23.6 (4016)
**Convenience of buying legal cannabis**				
More convenient	34.3 (1837)	40.0 (2037)	47.8 (2848)	41.1 (6722)
No difference	25.9 (1179)	26.9 (1144)	25.9 (1405)	26.2 (3728)
Less convenient	22.2 (953)	15.0 (569)	10.9 (549)	15.7 (2071)
Don’t know	17.6 (847)	18.1 (876)	15.5 (961)	17.0 (2684)
**Safety of using legal cannabis**				
Safer to use	42.6 (2144)	42.8 (2141)	46.8 (2806)	44.2 (7091)
No difference	34.5 (1602)	34.8 (1462)	33.1 (1774)	34.1 (4838)
Less safe to use	4.4 (180)	4.4 (158)	3.6 (177)	4.1 (515)
Don’t know	18.5 (890)	18.0 (859)	16.5 (1015)	17.6 (2764)
**Safety of buying legal cannabis**				
Safer to buy	49.8 (2520)	49.7 (2458)	54.0 (3213)	51.3 (8191)
No difference	29.7 (1362)	31.4 (1308)	29.0 (1549)	30.0 (4219)
Less safe to buy	4.1 (171)	3.0 (111)	3.5 (164)	3.5 (446)
Don’t know	16.3 (763)	15.9 (753)	13.5 (844)	15.1 (2360)

Weighted %, unweighted n. Sample sizes do not equate total due to missing data in variables: quality of legal products (*n* = 101); price of legal products (*n* = 92); convenience of buying legal products (*n* = 106); safety of using legal products (*n* = 103); and safety of buying legal products (*n* = 95)

After adjusting for covariates in the regression analysis, respondents in 2021 had higher odds of reporting that legal cannabis was of higher quality, no different, or didn’t know (vs. lower quality) than illegal cannabis compared to 2019 and 2020 (Table 3). Respondents in Quebec had higher odds of reporting legal cannabis was of higher quality or no different (vs. lower quality) compared to all other provinces except Prince Edward Island (odds ratio [OR] reversed from what is displayed in Table 3). Compared to less than monthly cannabis consumers, daily/almost daily consumers had higher odds of reporting that legal cannabis was of lower quality (vs. higher quality, no difference, or don’t know; OR reversed). Results focusing on ‘No difference’ and ‘Don’t know’ can be found in Table 3.

**Table 3:** Weighted multinomial logistic regression analysis for correlates of legal cannabis product perceptions, 2019–2021

	**Quality of legal cannabis products** **AOR (95% CI)** ***n* = 15,061**			**Price of legal cannabis products** **AOR (95% CI)** ***n* = 15,068**			**Convenience of buying legal cannabis products** **AOR (95% CI)** ***n* = 15,056**		
	**Higher quality** (vs. low)	**No diff** (vs. low)	**Don’t know** (vs. low)	**Less expensive** (vs. more)	**No diff** (vs. more)	**Don’t know** (vs. more)	**More convenient** (vs. less)	**No diff** (vs. less)	**Don’t know** (vs. less)
**Survey year**									
2021 (vs 2020)	**1.37 (1.17,1.61)**	**1.24 (1.06,1.45)**	**1.18 (1.01,1.38)**	**1.27 (1.05,1.54)**	**1.23 (1.06,1.42)**	1.07 (0.94,1.21)	**1.77 (1.50,2.09)**	**1.39 (1.16,1.66)**	**1.29 (1.06,1.56)**
2020 (vs 2019)	1.05 (0.89,1.24)	1.13 (0.96,1.34)	1.02 (0.87,1.20)	**1.49 (1.18,1.87)**	**1.44 (1.23,1.69)**	**1.28 (1.12,1.46)**	**1.74 (1.49,2.04)**	**1.54 (1.30,1.82)**	**1.57 (1.31,1.90)**
2021 (vs 2019)	**1.44 (1.23,1.68)**	**1.41 (1.21,1.64)**	**1.20 (1.03,1.40)**	**1.89 (1.54,2.32)**	**1.77 (1.54,2.05)**	**1.36 (1.21,1.54)**	**3.09 (2.65,3.60)**	**2.13 (1.81,2.51)**	**2.03 (1.69,2.43)**
**Province of residence**									
Québec	REF	REF	REF	REF	REF	REF	REF	REF	REF
British Columbia	**0.39 (0.31,0.49)**	**0.55 (0.44,0.70)**	0.82 (0.65,1.03)	**0.57 (0.43,0.76)**	**0.60 (0.49,0.74)**	1.15 (0.96,1.37)	**0.80 (0.64,0.98)**	1.00 (0.80,1.27)	1.29 (0.99,1.67)
Alberta	**0.43 (0.34,0.54)**	**0.52 (0.42,0.66)**	0.84 (0.67,1.06)	0.79 (0.59,1.05)	**0.63 (0.51,0.78)**	**1.27 (1.06,1.50**)	**2.00 (1.57,2.55)**	**1.54 (1.18,2.01)**	**2.03 (1.52,2.71)**
Saskatchewan	**0.50 (0.36,0.69)**	**0.63 (0.45,0.87)**	0.96 (0.70,1.33)	**0.53 (0.35,0.81)**	**0.57 (0.43,0.76)**	0.95 (0.74,1.22)	**1.68 (1.16,2.44)**	**1.56 (1.05,2.30)**	**1.99 (1.32,3.00)**
Manitoba	**0.47 (0.34,0.66)**	**0.70 (0.51,0.97)**	1.08 (0.79,1.50)	0.69 (0.46,1.05)	**0.54 (0.40,0.73)**	1.14 (0.90,1.45)	1.33 (0.96,1.85)	1.09 (0.76,1.55)	**1.57 (1.08,2.28)**
Ontario	**0.55 (0.45,0.67)**	**0.61 (0.50,0.75)**	**0.80 (0.64,0.99)**	1.06 (0.84,1.34)	**0.76 (0.64,0.91)**	1.12 (0.96,1.32)	**0.63 (0.52,0.75)**	**0.81 (0.66,0.99)**	0.99 (0.79,1.24)
New Brunswick	**0.55 (0.41,0.76)**	**0.64 (0.47,0.87)**	0.95 (0.69,1.29)	**0.51 (0.33,0.77)**	**0.60 (0.45,0.80)**	0.82 (0.64,1.04)	1.23 (0.88,1.72)	**1.57 (1.11,2.21)**	1.37 (0.93,2.02)
Nova Scotia	**0.27 (0.20,0.36)**	**0.52 (0.40,0.69)**	**0.65 (0.48,0.86)**	**0.59 (0.40,0.87)**	**0.61 (0.46,0.80**)	1.00 (0.79,1.27)	0.81 (0.61,1.08)	1.10 (0.82,1.48)	1.05 (0.75,1.48)
Prince Edward Island	0.65 (0.33,1.25)	0.81 (0.41,1.61)	0.98 (0.52,1.88)	1.51 (0.72,3.20)	0.81 (0.45,1.46)	**1.78 (1.03,3.07)**	**2.69 (1.13,6.38)**	1.88 (0.74,4.78)	2.04 (0.77,5.40)
Newfoundland & Labrador	**0.40 (0.28,0.57)**	**0.59 (0.41,0.83)**	0.76 (0.55,1.06)	1.46 (0.95,2.24)	0.81 (0.58,1.13)	1.26 (0.96,1.64)	**1.93 (1.24,3.02)**	**1.67 (1.04,2.71)**	**2.00 (1.21,3.33)**
**Cannabis use frequency**									
Past year, but less than monthly	REF	REF	REF	REF	REF	REF	REF	REF	REF
Monthly	0.82 (0.67,1.01)	1.06 (0.86,1.30)	**0.45 (0.37,0.55)**	**1.34 (1.04,1.72)**	**1.28 (1.08,1.52)**	**0.53 (0.46,0.61)**	**0.65 (0.53,0.79)**	0.92 (0.74,1.14)	**0.43 (0.35,0.54)**
Weekly	0.81 (0.66,1.00)	0.84 (0.68,1.04)	**0.30 (0.25,0.37)**	1.24 (0.95,1.62)	1.16 (0.97,1.39)	**0.36 (0.31,0.42)**	**0.67 (0.54,0.82)**	0.93 (0.74,1.15)	**0.31 (0.25,0.40**)
Daily/almost daily	**0.48 (0.40,0.57)**	**0.54 (0.45,0.64)**	**0.17 (0.14,0.20)**	1.15 (0.91,1.44)	**0.85 (0.72,0.99)**	**0.25 (0.22,0.28)**	**0.43 (0.36,0.51)**	**0.70 (0.58,0.84)**	**0.21 (0.17,0.25)**

Models were adjusted for age, gender, ethnicity/race, education, income adequacy, and survey device used

### Price of legal cannabis

Across all years, close to half of respondents reported that legal cannabis was more expensive than illegal cannabis, one-fifth reported there was no difference, and close to one in ten reported legal cannabis was less expensive (Table 2; Additional File 1b).

After adjusting for covariates in the regression analysis, respondents in 2019 had higher odds of reporting that legal cannabis was more expensive than respondents in 2020 and 2021 (vs. less expensive, no difference, don’t know; OR reversed); and respondents in 2020 had higher odds of reporting it as more expensive than respondents in 2021 (vs. less expensive, no difference). Respondents in Québec had higher odds of reporting that legal cannabis was less expensive than illegal cannabis compared to respondents in British Columbia, Saskatchewan, New Brunswick, and Nova Scotia (vs. more expensive; OR reversed). Compared to less than monthly consumers, monthly consumers had higher odds of reporting legal cannabis was less expensive or did not differ to illegal cannabis (vs. more expensive). Results focusing on ‘No difference’ and ‘Don’t know’ can be found in Table 3.

### Convenience of buying legal cannabis

A greater percentage of respondents reported that legal cannabis was more convenient than illegal cannabis in all three years (34–48%), one-quarter reported there was no difference, and one-sixth reported legal cannabis was less convenient (Table 2; Additional File 1c).

After adjusting for covariates in the regression analysis, respondents in 2021 and 2020 had higher odds of reporting legal cannabis was more convenient to buy than illegal cannabis compared to in 2019, and respondents in 2021 had higher odds than respondents in 2020 (vs. less convenient). Compared to respondents in Québec, respondents in Alberta, Saskatchewan, Prince Edward Island, and Newfoundland and Labrador had higher odds of reporting legal cannabis was more convenient to buy (vs. less convenient). Respondents in Québec had higher odds of reporting legal cannabis was more convenient to buy than respondents in Ontario and British Columbia (vs. less convenient; OR reversed). Less than monthly consumers had higher odds of reporting legal cannabis was more convenient to buy than more frequent consumers (vs. less convenient; OR reversed). Results focusing on ‘No difference’ and ‘Don’t know’ can be found in Table 3.

### Safety of consuming legal cannabis

Across all years, close to half of respondents reported that legal cannabis was safer to use than illegal cannabis, one-third reported there was no difference, and one in twenty reported legal cannabis was less safe to use (Table 2; Additional File 1d).

After adjusting for covariates in the regression analysis, respondents in 2021 had higher odds of reporting that it was safer to use legal cannabis compared to 2019 and 2020 (vs. less safe; Table 4). There were no significant associations between province of residence and perceived safety of using legal cannabis. Less than monthly consumers had higher odds of reporting it was safer to use legal cannabis than monthly and daily/almost daily consumers (vs. less safe; OR reversed). Results focusing on ‘No difference’ and ‘Don’t know’ can be found in Table 4.

**Table 4:** Weighted multinomial logistic regression analysis for correlates of legal cannabis product safety perceptions, 2019–2021

	**Safer to use legal cannabis products** **AOR (95% CI)** ***N* = 15,060**			**Safer purchasing legal cannabis products** **AOR (95% CI)** ***N* = 15,067**		
	**Safer to use** (vs. less)	**No diff** (vs. less)	**Don’t know** (vs. less)	**Safer to buy** (vs. less)	**No diff** (vs. less)	**Don’t know** (vs. less)
**Survey year**						
2021 (vs 2020)	**1.42 (1.07, 1.89)**	1.19 (0.89, 1.59)	1.20 (0.89, 1.62)	0.98 (0.73, 1.33)	0.81 (0.60, 1.10)	0.78 (0.56, 1.07)
2020 (vs 2019)	0.97 (0.73, 1.30)	0.96 (0.72, 1.28)	0.95 (0.70, 1.29)	1.33 (0.98, 1.80)	**1.38 (1.01, 1.88)**	1.32 (0.95, 1.83)
2021 (vs 2019)	**1.39 (1.06, 1.82)**	1.14 (0.87, 1.50)	1.15 (0.86, 1.53)	1.31 (0.99, 1.73)	1.12 (0.85, 1.49)	1.02 (0.76, 1.38)
**Province of residence**						
British Columbia	0.79 (0.54, 1.15)	1.21 (0.83, 1.78)	1.47 (0.98, 2.21)	1.19 (0.79, 1.81)	**2.07 (1.36, 3.16)**	**2.03 (1.30, 3.16)**
Alberta	0.97 (0.64, 1.46)	0.97 (0.63, 1.48)	1.24 (0.80, 1.94)	1.16 (0.75, 1.79)	1.11 (0.71, 1.73)	1.31 (0.82, 2.10)
Saskatchewan	0.99 (0.55, 1.77)	1.20 (0.67, 2.16)	1.20 (0.65, 2.22)	1.61 (0.83, 3.10)	1.78 (0.91, 3.51)	1.81 (0.90, 3.62)
Manitoba	0.93 (0.50, 1.75)	1.02 (0.54, 1.92)	1.31 (0.68, 2.53)	1.07 (0.59, 1.96)	1.14 (0.62, 2.10)	1.38 (0.72, 2.63)
Ontario	0.74 (0.53, 1.04)	0.85 (0.60, 1.21)	1.11 (0.77, 1.60)	0.92 (0.64, 1.30)	1.16 (0.81, 1.66)	1.31 (0.90, 1.92)
New Brunswick	1.02 (0.59, 1.77)	1.17 (0.68, 2.04)	1.54 (0.87, 2.73)	1.75 (0.87, 3.50)	**2.11 (1.04, 4.27)**	**2.38 (1.16, 4.91)**
Nova Scotia	0.83 (0.45, 1.53)	1.28 (0.69, 2.38)	1.29 (0.68, 2.46)	1.69 (0.85, 3.56)	**2.39 (1.19, 4.79)**	2.01 (0.99, 4.11)
Prince Edward Island	0.48 (0.17, 1.32)	0.40 (0.14, 1.18)	0.48 (0.16, 1.48)	1.39 (0.41, 4.67)	1.27 (0.37, 4.39)	1.68 (0.43, 6.49)
Newfoundland & Labrador	1.29 (0.66, 2.52)	1.25 (0.63, 2.46)	1.43 (0.70, 2.89)	**4.36 (1.70, 11.19)**	**4.13 (1.59, 10.69)**	**3.97 (1.50, 10.56)**
Québec	REF	REF	REF	REF	REF	REF
**Cannabis use frequency**						
Past year but less than monthly	REF	REF	REF	REF	REF	REF
Monthly	**0.59 (0.41, 0.83)**	0.88 (0.62, 1.26)	**0.45 (0.31, 0.64)**	**0.58 (0.41, 0.82)**	0.86 (0.60, 1.24)	**0.41 (0.28, 0.59)**
Weekly	0.73 (0.50, 1.06)	1.17 (0.80, 1.71)	**0.38 (0.26, 0.57)**	0.78 (0.53, 1.15)	1.28 (0.86, 1.91)	**0.42 (0.28, 0.63)**
Daily/almost daily	**0.49 (0.36, 0.69)**	1.32 (0.95, 1.85)	**0.33 (0.23, 0.46)**	**0.61 (0.43, 0.88)**	**1.47 (1.02, 2.11)**	**0.35 (0.24, 0.50)**

Models were adjusted for age, gender, ethnicity/race, education, income adequacy, and survey device used

### Safety of buying cannabis

In all three survey years, one-half of respondents reported that legal cannabis was safer to buy than illegal cannabis, close to one-third reported there was no difference, and one in twenty reported legal cannabis was less safe to buy (Table 2; Additional File 1e).

After adjusting for covariates in the regression analysis, respondents in Newfoundland and Labrador had higher odds of reporting it was safer to buy legal than illegal cannabis compared to respondents in Québec (vs. less safe). Respondents in British Columbia, New Brunswick, Nova Scotia, and Newfoundland and Labrador had higher odds of reporting that there was no difference regarding the safety of legal cannabis compared to respondents in Québec (vs. less safe). Less than monthly consumers had higher odds of reporting legal cannabis was safer to buy compared to monthly and daily/almost daily consumers (vs. less safe; OR reversed). Results focusing on ‘No difference’ and ‘Don’t know’ can be found in Table 4.

The overall interaction between survey year and cannabis use frequency was not significant for any perceptions modelled in this study (Quality: *F* = 1.26, *p* = 0.200; Price: *F* = 1.43, *p* = 0.105; Convenience of buying: *F* = 0.88, *p* = 0.599; Safety of using: *F* = 1.05, *p* = 0.404; Safety of buying: *F* = 1.26, *p* = 0.197) and therefore not presented in the main analysis (Additional File 2).

## Discussion

In the three years since legalization, Canadian cannabis consumers had increasingly favourable perceptions of legal cannabis products compared to illegal cannabis products regarding quality, convenience, and safety, but to a lesser extent for price, mirroring the results of a similar study examining perceptions of legal products conducted in U.S. states with non-medical cannabis markets [27]. Although most consumers perceived legal cannabis to be more expensive than illegal cannabis, perceptions of price improved over the three years, as did all other perceptions measured. These results are consistent with recent research and industry data demonstrating that the price of legal cannabis has decreased since legalization [2–4, 8, 11]. Given that the price differential between illegal and legal cannabis narrowed in the first three years post-legalization, consumers may have increasingly favourable perceptions of legal cannabis prices in the future [8, 34]. Future studies should examine the implications of decreasing legal cannabis prices: although lower prices may help displace illegal sources, research on the price elasticity of cannabis demonstrates that consumption and initiation increase as price decreases [35, 36]. Therefore, to achieve the public health objectives of the Cannabis Act, pricing strategies will need to consider both the benefits and risks of lower cannabis prices [1].

A safe and regulated cannabis supply is a key objective of the Cannabis Act. The current study indicates that most consumers perceive legal cannabis to be safer to buy and use than illegal cannabis; in contrast, fewer than one in 20 respondents reported that legal cannabis was less safe to use and buy. Acquiring illegal substances commonly comes with a certain level of risk: risk of criminal sanctions and uncertainty regarding product contents, including potency and potential contaminants. Unlike illegally sourced products, legal cannabis is subject to testing and product standards that minimize contaminants [37, 38]. Moreover, legal cannabis products must display THC content and other product information, including ingredients and nutritional information for edible products [9]. In 2022, the federal government launched a public education campaign to highlight the greater safety and product standards for legal cannabis [39]. However, governments must ensure that promoting legal cannabis as ‘safer’ than illegal cannabis does not equal ‘safe’ and encourage non-consumers to initiate use. Regarding the safety of purchasing cannabis, compared to respondents in Québec, respondents in British Columbia, New Brunswick, Nova Scotia, and Newfoundland & Labrador perceived legal cannabis as “no different” to buy than illegal cannabis. This likely reflects the extent to which illegal cannabis sources were established prior to legalization in Canada [40]. This was particularly the case in British Columbia, in which previously illegal retail stores were able to apply for legal licenses, and local governments had licensed cannabis dispensaries prior to legalization.

Consumers report that legal cannabis was increasingly convenient to buy across the three years of study. Consumer perceptions mirror the increase in use of legal retail stores as well as the increase in the number of legal retail stores over time across Canada [4]. However, there are large differences in the number of legal stores per capita across provinces, and this is reflected in perceptions of convenience across the provinces in the current study [24, 25, 41, 42]. In general, respondents in provinces with a higher number of stores per capita were more likely to report that legal cannabis was more convenient to buy than illegal cannabis compared to respondents in Québec, the province with the lowest number of stores per capita in 2020 and 2021. Only respondents in British Columbia and Ontario were less likely to report that legal cannabis was more convenient to buy, potentially because Ontario had only recently increased its stores per capita. Indeed, Ontario had the lowest stores per capita in 2019 due to delays in licensing and store openings as well as a restrictive cap on the number of licenses issued. Similar to price, retail policies must strike a balance whereby legal cannabis is more convenient to buy than illegal but does not encourage uptake or initiation from non-consumers. Recent research has demonstrated promise in achieving this balance; however, this research was conducted prior to the introduction of edibles, topicals, and extracts and Ontario’s increase in retail stores [24].

In general, cannabis consumers in Québec had favourable perceptions of legal cannabis with regard to quality and price. Indeed, Québec has had some of the lowest legal cannabis prices since legalization [12, 40, 43, 44]. One could expect that Québec respondents would perceive legal cannabis products as less convenient because Québec has the lowest number of stores per capita across all provinces and has additional restrictions on cannabis products (a THC limit of 30% across all products). However, respondents in only four provinces were more likely to perceive cannabis products as more convenient to buy than respondents in Québec. This could be due to the government strategically selecting store locations that would maximize access to stores across the population – Québec is the most populated province with a government-run retail structure – compared to private-run retail structures where retail stores may be concentrated in urban settings [44]. The current study did not find any consistent evidence that Québec’s more restrictive retail market has translated into unfavourable perceptions of legal cannabis compared to other provinces.

Perceptions of cannabis varied by frequency of cannabis consumption. In the present study, daily or almost daily consumers had less favourable perceptions of the quality, price, convenience, and safety of legal cannabis. Less favourable perceptions for daily consumers may be due to the established relationships with prior illegal sources: frequent consumers may have a stronger relationship with and trust their illegal source [26]. Frequent consumers may also purchase cannabis in larger quantities from illegal sources than legal retail sources permit (30 g of dried flower or non-flower equivalent), which may result in lower prices through quantity discounts [1, 45–47]. Indeed, recent research has demonstrated that price is one of the most important factors in determining whether consumers purchase illegally or legally [45–47]. There is a public health conflict regarding daily consumers, whereby governments need to increase perceptions of legal cannabis to transition the more frequent consumers to the legal market, while also discouraging frequent consumption due to negative health consequences of such use [14, 20].

### Limitations

This study is subject to limitations common to survey research. Respondents were recruited using non-probability-based sampling; therefore, the findings do not necessarily provide nationally representative estimates. The data were weighted by age group, sex, region, education, and tobacco smoking status in Canada. Cannabis use estimates were generally lower than national estimates for young adults, and higher than national surveys in Canada. This is likely because the ICPS sampled individuals aged 16–65, whereas national surveys included older adults, who are known to have lower rates of cannabis use [48].

Perceptions may vary depending on the cannabis product in question. The current study did not ask perceptions of individual cannabis products (i.e., dried flower, edibles) and not all products were available to purchase from legal retail stores from October 2018. Dried flower and some oils were available in October 2018, and all other products were available to purchase from December 2019. However, dried flower is still the most used product and may represent a large proportion of perceptions [3, 4, 49].

## Conclusions

In the three years since legalization, Canadian cannabis consumers generally had increasingly favourable perceptions of legal cannabis products relative to illegal cannabis products, with variation across the provinces and frequency of cannabis consumption. The perceived cost of cannabis is particularly important given that it is a primary reason for purchasing from illegal versus legal markets [3, 4, 26]. Three years after legalization in Canada, the price of legal cannabis was still perceived to be more expensive than illegal cannabis; however, perceptions had begun to shift as legal retail prices declined in 2020 and 2021. Findings from this study will help government, policymakers, and public health researchers to better understand consumers perceptions of legal cannabis. Future studies should examine longer-term changes in consumer perceptions of legal cannabis in Canada, as well as the optimal policy mix to meet the dual objectives of the *Cannabis Act* in terms of displacing the illegal market without promoting greater cannabis use among Canadians.

## Supplementary Information

**Additional file 1.**

**Additional file 2.**

## Data Availability

The data that support the findings of this study are available from the International Cannabis Policy Study (www.cannabisproject.ca) but restrictions apply to the availability of these data, and so are not publicly available. Data are however available from the authors upon reasonable request – please contact David Hammond (david.hammond@uwaterloo.ca).

## References

[1] Government of Canada. Justice Laws Website: Cannabis Act (S.C. 2018, c.16)

[2] Health Canada. Canadian Cannabis Survey 2019 – Summary. https://www.canada.ca/en/health-canada/services/publications/drugs-health-products/canadian-cannabis-survey-2019-summary.html. Accessed July 5, 2022.

[3] Health Canada. Canadian Cannabis Survey 2020: Summary. https://www.canada.ca/en/health-canada/services/drugs-medication/cannabis/research-data/canadian-cannabis-survey-2020-summary.html. Accessed July 5, 2022.

[4] Health Canada. Canadian Cannabis Survey 2021: Summary. https://www.canada.ca/en/health-canada/services/drugs-medication/cannabis/research-data/canadian-cannabis-survey-2021-summary.html#a2. Accessed July 5, 2022.

[5] Rotermann M (2021). Looking back from 2020, how cannabis use and related behaviours changed in Canada. Health Rep.

[6] Statistics Canada. Retail trade sales by province and territory (x 1,000). Table: 20–10–0008–01 (formerly CANSIM 080–0020). Author: Ottawa, ON. https://www150.statcan.gc.ca/t1/tbl1/en/tv.action?pid=2010000801) Accessed June 30, 2022.

[7] Armstrong MJ, Cantor N, Smith BT, Jesseman R, Hobin E, Myran DT (2022). Interrupted time series analysis of Canadian legal cannabis sales during the COVID-19 pandemic. Drug Alcohol Rev.

[8] Wadsworth E, Driezen P, Pacula RL, Hammond D (2022). Cannabis flower prices and transitions to legal sources after legalization in Canada. Drug Alcohol Depend.

[9] Health Canada. Final regulations: Edible cannabis, cannabis extracts, cannabis topicals. https://www.canada.ca/en/health-canada/services/drugs-medication/cannabis/resources/regulations-edible-cannabis-extracts-topicals.html) Accessed June 20, 2022

[10] Québec Government. The cannabis regulation act. https://encadrementcannabis.gouv.qc.ca/en/loi/loi-encadrant-le-cannabis/ Accessed July 5, 2022.

[11] Ontario Cannabis Store. 2020–2021 Annual Report. https://www.doingbusinesswithocs.ca/wp-content/uploads/2022/02/OCS-2020-21-Annual-Report_ENG.pdf) Accessed June 21, 2022.

[12] Mahamad S, Wadsworth E, Rynard V, Goodman S, Hammond D (2020). Availability, retail price and potency of legal and illegal cannabis in Canada after recreational cannabis legalisation. Drug Alcohol Rev.

[13] National Academies of Sciences Engineering and Medicine (2017). The Health Effects of Cannabis and Cannabinoids: The Current State of Evidence and Recommendations for Research.

[14] Hall W (2015). What has research over the past two decades revealed about the adverse health effects of recreational cannabis use?. Addiction.

[15] van der Pol P, Liebregts N, de Graaf R, Korf DJ, van den Brink W, van Laar M (2013). Predicting the transition from frequent cannabis use to cannabis dependence: a three-year prospective study. Drug Alcohol Depend.

[16] Di Forti M, Quattrone D, Freeman TP, Tripoli G, Gayer-Anderson C, Quigley H (2019). The contribution of cannabis use to variation in the incidence of psychotic disorder across Europe (EU-GEI): a multicentre case-control study. Lancet Psychiat.

[17] Moore TH, Zammit S, Lingford-Hughes A, Barnes TR, Jones PB, Burke M (2007). Cannabis use and risk of psychotic or affective mental health outcomes: a systematic review. Lancet.

[18] Degenhardt L, Coffey C, Romaniuk H, Swift W, Carlin JB, Hall WD (2013). The persistence of the association between adolescent cannabis use and common mental disorders into young adulthood. Addiction.

[19] Manrique-Garcia E, Zammit S, Dalman C, Hemmingsson T, Andreasson S, Allebeck P (2012). Cannabis, schizophrenia and other non-affective psychoses: 35 years of follow-up of a population-based cohort. Psychol Med.

[20] Hall W, Degenhardt L (2014). The adverse health effects of chronic cannabis use. Drug Test Anal.

[21] Meier MH, Caspi A, Ambler A, Harrington H, Houts R, Keefe RS (2012). Persistent cannabis users show neuropsychological decline from childhood to midlife. Proc Natl Acad Sci U S A.

[22] Shi Y, Lenzi M, An R (2015). Cannabis Liberalization and Adolescent Cannabis Use: A Cross-National Study in 38 Countries. PLoS ONE.

[23] Health Canada. Authorized cannabis retailers in the provinces and territories. https://www.canada.ca/en/health-canada/services/drugs-medication/cannabis/laws-regulations/provinces-territories.html Accessed September 16, 2022.

[24] Armstrong MJ (2021). Relationships between increases in Canadian cannabis stores, sales, and prevalence. Drug Alcohol Depend.

[25] Canadian Centre on Substance Use and Addiction. Policy and Regulations (Cannabis). https://www.ccsa.ca/policy-and-regulations-cannabis) Accessed June 30, 2022.

[26] Goodman S, Wadsworth E, Hammond D (2022). Reasons for purchasing cannabis from illegal sources in legal markets: findings among cannabis consumers in Canada and US states, 2019–2020. J Studies Alcohol Drugs.

[27] Fataar F, Goodman S, Wadsworth E, Hammond D (2021). Consumer perceptions of ‘legal’and ‘illegal’cannabis in US states with legal cannabis sales. Addict Behav.

[28] Corsetti D, Burkhalter R, Hammond D (2022). International Cannabis Policy Study Technical Report - Wave 4 (2021).

[29] Goodman S, Burkhalter R, Hammond D (2020). International Cannabis Policy Study Technical Report - Wave 2 (2019).

[30] Goodman S, Burkhalter R, Hammond D (2021). International Cannabis Policy Study Technical Report - Wave 3 (2020).

[31] Hammond D, Goodman S, Wadsworth E, Rynard V, Boudreau C, Hall W (2020). Evaluating the impacts of cannabis legalization: the International Cannabis Policy Study. Int J Drug Policy.

[32] Greaves L, Ritz SA (2020). Sex, gender and health: Mapping the landscape of research and policy. Int J Environ Res Public Health.

[33] American Association for Public Opinion Research. Standard definitions: Final dispositions of case codes and outcome rates for surveys. (https://www.aapor.org/AAPOR_Main/media/publications/Standard-Definitions20169theditionfinal.pdf). Accessed June 21, 2022.

[34] Statistics Canada. StatsCannabis data availability: Crowdsourced cannabis prices, fourth quarter 2019. https://www150.statcan.gc.ca/n1/daily-quotidien/200123/dq200123c-eng.html. Accessed June 1, 2022.

[35] Pacula RL, Lundberg R (2013). Why changes in price matter when thinking about marijuana policy: a review of the literature on the elasticity of demand. Public Health Rev.

[36] Vincent PC, Collins RL, Liu L, Yu J, De Leo JA, Earleywine M (2017). The effects of perceived quality on behavioral economic demand for marijuana: A web-based experiment. Drug Alcohol Depend.

[37] Health Canada. Mandatory cannabis testing for pesticide active ingredients – Requirements. https://www.canada.ca/en/public-health/services/publications/drugs-health-products/cannabis-testing-pesticide-requirements.html Accessed 16 Sept 2022.

[38] Health Canada. Guidance document: Good production practices guide for cannabis. https://www.canada.ca/en/health-canada/services/cannabis-regulations-licensed-producers/good-production-practices-guide/guidance-document.html Accessed 16 Sept 2022

[39] Government of Canada. Reduce your risk: Choose legal cannabis. (https://www.canada.ca/en/health-canada/services/drugs-medication/cannabis/personal-use/reduce-risk-choose-legal.html?utm_medium=email&_hsmi=212263516&_hsenc=p2ANqtz-_Jh9eUtH7y6TnOsZ53ceu2Fx_WQHk7OT4B359rrtytd_wbwYDV6fjZNhNKo6LiIwH_iMy5b9S_lwFHATxwZDM5A-I6tQ&utm_content=212263516&utm_source=hs_email) Accessed July 5, 2022.

[40] Mahamad S, Hammond D (2019). Retail price and availability of illicit cannabis in Canada. Addict Behav.

[41] Myran DT, Staykov E, Cantor N, Taljaard M, Quach BI, Hawken S, Tanuseputro P (2022). How has access to legal cannabis changed over time? An analysis of the cannabis retail market in Canada 2 years following the legalisation of recreational cannabis. Drug Alcohol Rev.

[42] Wadsworth E, Driezen P, Hammond D (2021). Retail availability and legal purchases of dried flower in Canada post-legalization. Drug Alcohol Depend.

[43] Wadsworth E, Driezen P, Goodman S, Hammond D (2020). Differences in self-reported cannabis prices across purchase source and quantity purchased among Canadians. Addict Res Theory.

[44] Gibbs B, Reed T, Wride S. Cannabis legalisation – Canada’s experience. A research report by Public First. (https://www.publicfirst.co.uk/wp-content/uploads/2021/10/REPORT-Cannabis-in-Canada-Public-First-October-2021.pdf) Accessed June 1, 2022.

[45] Caulkins JP, Padman R (1993). Quantity discounts and quality premia for illicit drugs. J Am Stat Assoc.

[46] Caulkins JP (2007). Price and purity analysis for illicit drug: data and conceptual issues. Drug Alcohol Depend.

[47] Clements KW (2006). Pricing and packaging: the case of marijuana. J Business.

[48] Canadian Institute for Health Information (2021). Cannabis Use in Canada [infographic].

[49] Goodman S, Wadsworth E, Leos-Toro C, Hammond D (2020). Prevalence and forms of cannabis use in legal vs. illegal recreational cannabis markets. Int J Drug Policy.

