# Supplementary material for: Consumer perceptions of legal cannabis products in Canada, 2019–2021: a repeat cross-sectional study
Source: BMC Public Health. 2022 Nov 8;22:2048. doi: 10.1186/s12889-022-14492-z (PMC9644504; doi:10.1186/s12889-022-14492-z)
Supplement: Supplementary file 1 — Additional file 1. [file 12889_2022_14492_MOESM1_ESM.docx]

**Additional File 1a:** **Perceptions of the quality of cannabis from legal sources, 2019-2021.**

**Additional File 1b: Perceptions of the price of cannabis from legal sources, 2019-2021.**

**Additional File 1c:** **Perceptions of the convenience of buying cannabis from legal sources, 2019-2021.**

**Additional File 1d:** **Perceptions of the safety of using cannabis from legal sources, 2019-2021.**

**Additional File 1e:** **Perceptions of the safety of buying cannabis from legal sources, 2019-2021.**
