# Supplementary material for: Consumer perceptions of legal cannabis products in Canada, 2019–2021: a repeat cross-sectional study
Source: BMC Public Health. 2022 Nov 8;22:2048. doi: 10.1186/s12889-022-14492-z (PMC9644504; doi:10.1186/s12889-022-14492-z)
Supplement: Supplementary file 2 — Additional file 2. [file 12889_2022_14492_MOESM2_ESM.docx]

**Additional File 2: Interaction results of weighted multinomial logistic regression analysis for correlates of legal cannabis product safety perceptions among past 12-month cannabis consumers of legal age to purchase cannabis, 2019-2021**

|  | **Quality of legal cannabis**  **AOR (95% CI)**  **n=15,061** | | | **Price of legal cannabis**  **AOR (95% CI)**  **n=15,068** | | |
| --- | --- | --- | --- | --- | --- | --- |
|  | **Higher quality**  (vs. low) | **No diff.**  (vs. low) | **Don’t know**  (vs. low) | **Less expensive**  (vs. more) | **No diff.**  (vs. more) | **Don’t know**  (vs. more) |
| **Past year, but less than monthly** |  |  |  |  |  |  |
| 2021 vs 2020 | 1.00 (0.70,1.43) | 0.99 (0.70,1.44) | 0.88 (0.63,1.22) | 1.07 (0.70,1.65) | **1.39 (1.04,1.87)** | 0.98 (0.80,1.19) |
| 2021 vs 2019 | 1.03 (0.75,1.42) | 1.18 (0.85,1.64) | 0.91 (0.67,1.23) | **1.62 (1.06,2.47)** | **1.80 (1.37,2.36)** | **1.21 (1.00,1.46)** |
| 2020 vs 2019 | 1.03 (0.72,1.46) | 1.18 (0.83,1.68) | 1.04 (0.75,1.43) | 1.50 (0.94,2.39) | 1.29 (0.95,1.74) | **1.24 (1.01,1.51)** |
| **Monthly** |  |  |  |  |  |  |
| 2021 vs 2020 | **1.73 (1.18,2.53)** | 1.18 (0.81,1.71) | 1.31 (0.89,1.92) | 1.08 (0.71,1.62) | 1.15 (0.85,1.57) | 1.18 (0.88,1.58) |
| 2021 vs 2019 | **1.73 (1.21,2.47)** | 1.31 (0.93,1.86) | 1.24 (0.87,1.77) | 1.44 (0.93,2.23) | **1.46 (1.09,1.96)** | 1.28 (0.97,1.70) |
| 2020 vs 2019 | 0.99 (0.68,1.48) | 1.12 (0.76,1.64) | 0.95 (0.65,1.40) | 1.34 (0.84,2.15) | 1.26 (0.92,1.74) | 1.09 (0.80,1.47) |
| **Weekly** |  |  |  |  |  |  |
| 2021 vs 2020 | 1.30 (0.89,1.90) | 1.29 (0.88,1.88) | 1.09 (0.74,1.59) | 1.12 (0.70,1.80) | 1.11 (0.80,1.53) | 0.95 (0.70,1.30) |
| 2021 vs 2019 | 1.02 (0.71,1.46) | 1.17 (0.81,1.68) | 1.18 (0.81,1.71) | 1.22 (0.78,1.90) | **1.59 (1.15,2.18)** | **1.73 (1.26,2.36)** |
| 2020 vs 2019 | 0.78 (0.53,1.17) | 0.91 (0.61,1.34) | 1.08 (0.72,1.62) | 1.09 (0.65,1.82) | **1.43 (1.00,2.05)** | **1.81 (1.28,2.55)** |
| **Daily/almost daily** |  |  |  |  |  |  |
| 2021 vs 2020 | **1.41 (1.10,1.79)** | **1.34 (1.05,1.71)** | **1.36 (1.04,1.76)** | **1.55 (1.14,2.11)** | 1.24 (0.96,1.59) | 1.17 (0.91,1.50) |
| 2021 vs 2019 | **1.76 (1.38,2.24)** | **1.63 (1.29,2.06)** | **1.32 (1.03,1.70)** | **3.05 (2.11,4.41)** | **2.12 (1.63,2.75)** | **1.46 (1.13,1.87)** |
| 2020 vs 2019 | 1.25 (0.96,1.63) | 1.21 (0.94,1.57) | 0.98 (0.74,1.28) | **1.97 (1.31,2.98)** | **1.71 (1.28,2.30)** | 1.24 (0.94,1.64) |
|  | **Convenience of buying legal cannabis products**  **AOR (95% CI)**  **n=15,056** | | | **Safer to use legal cannabis products**  **AOR (95% CI)**  **N=15,060** | | |
|  | **More convenient**  (vs. less) | **No diff.**  (vs. less) | **Don’t know**  (vs. less) | **Safer to use**  (vs. less) | **No diff.**  (vs. less) | **Don’t know**  (vs. less) |
| **Past year, but less than monthly** |  |  |  |  |  |  |
| 2021 vs 2020 | **1.75 (1.21,2.52)** | **1.56 (1.04,2.34)** | 1.28 (0.87,1.89) | 1.06 (0.56,2.00) | 1.04 (0.54,2.01) | 0.86 (0.45,1.66) |
| 2021 vs 2019 | **3.09 (2.26,4.21)** | **2.13 (1.51,3.01)** | **2.09 (1.50,2.91)** | 0.99 (0.57,1.76) | 1.07 (0.59,1.92) | 0.94 (0.52,1.68) |
| 2020 vs 2019 | **1.77 (1.27,2.46)** | 1.36 (0.95,1.97) | **1.63 (1.15,2.31)** | 0.94 (0.50,1.78) | 1.02 (0.54,1.95) | 1.09 (0.57,2.06) |
| **Monthly** |  |  |  |  |  |  |
| 2021 vs 2020 | **1.86 (1.28,2.70)** | 1.19 (0.80,1.77) | 1.54 (0.99,2.41) | **1.99 (1.12,3.55)** | 1.43 (0.78,2.58) | **1.92 (1.02,3.60)** |
| 2021 vs 2019 | **2.48 (1.76,3.49)** | **1.86 (1.29,2.69)** | **1.86 (1.23,2.82)** | 1.48 (0.82,2.67) | 1.20 (0.66,2.19) | 1.16 (0.62,2.17) |
| 2020 vs 2019 | 1.33 (0.94,1.89) | **1.56 (1.07,2.27)** | 1.21 (0.79,1.85) | 0.74 (0.41,1.34) | 0.84 (0.46,1.55) | 0.60 (0.32,1.14) |
| **Weekly** |  |  |  |  |  |  |
| 2021 vs 2020 | 1.36 (0.91,2.04) | 1.02 (0.66,1.48) | 0.90 (0.55,1.47) | 0.89 (0.44,1.80) | 0.71 (0.34,1.46) | 0.70 (0.33,1.50) |
| 2021 vs 2019 | **2.89 (2.02,4.13)** | **1.95 (1.33,2.86)** | **2.21 (1.39,3.49)** | 1.18 (0.65,2.15) | 0.84 (0.46,1.53) | 0.91 (0.47,1.75) |
| 2020 vs 2019 | **2.13 (1.44,3.14)** | **1.91 (1.26,2.90)** | **2.44 (1.51,3.96)** | 1.34 (0.65,2.77) | 1.19 (0.58,2.48) | 1.29 (0.60,2.81) |
| **Daily/almost daily** |  |  |  |  |  |  |
| 2021 vs 2020 | **1.92 (1.48,2.49)** | **1.57 (1.19,2.07)** | 1.33 (0.95,1.87) | 1.65 (1.04,2.61) | 1.41 (0.90,2.23) | 1.42 (0.86,2.35) |
| 2021 vs 2019 | **3.54 (2.76,4.53)** | **2.33 (1.80,3.01)** | **1.87 (1.35,2.58)** | 1.83 (1.18,2.84) | 1.37 (0.89,2.11) | 1.39 (0.87,2.24) |
| 2020 vs 2019 | **1.84 (1.42,2.39)** | **1.49 (1.14,1.94)** | 1.40 (0.99,1.98) | 1.11 (0.71,1.74) | 0.97 (0.62,1.50) | 0.98 (0.60,1.61) |
|  | **Safer purchasing legal cannabis products**  **AOR (95% CI)**  **N=15,067** | | |  |  |  |
|  | **Safer to buy**  (vs. less) | **No diff.**  (vs. less) | **Don’t know**  (vs. less) |  |  |  |
| **Past year, but less than monthly** |  |  |  |  |  |  |
| 2021 vs 2020 | 0.94 (0.48,1.85) | 0.81 (0.40,1.63) | 0.67 (0.34,1.36) |  |  |  |
| 2021 vs 2019 | 0.89 (0.50,1.60) | 0.79 (0.43,1.46) | 0.67 (0.36,1.23) |  |  |  |
| 2020 vs 2019 | 0.95 (0.49,1.84) | 0.98 (0.49,1.95) | 0.99 (0.50,1.96) |  |  |  |
| **Monthly** |  |  |  |  |  |  |
| 2021 vs 2020 | 0.97 (0.51,1.83) | 0.60 (0.31,1.15) | 0.96 (0.48,1.93) |  |  |  |
| 2021 vs 2019 | 1.66 (0.96,2.89) | 1.60 (0.90,2.85) | 1.38 (0.75,2.52) |  |  |  |
| 2020 vs 2019 | 1.71 (0.94,3.12) | **2.68 (1.44,5.00)** | 1.44 (0.74,2.79) |  |  |  |
| **Weekly** |  |  |  |  |  |  |
| 2021 vs 2020 | 0.89 (0.44,1.77) | 0.86 (0.42,1.75) | 0.66 (0.31,1.40) |  |  |  |
| 2021 vs 2019 | 1.57 (0.81,3.03) | 1.36 (0.70,2.66) | 1.36 (0.65,2.81) |  |  |  |
| 2020 vs 2019 | 1.77 (0.88,3.52) | 1.58 (0.78,3.21) | 2.07 (0.97,4.42) |  |  |  |
| **Daily/almost daily** |  |  |  |  |  |  |
| 2021 vs 2020 | 1.06 (0.65,1.74) | 0.92 (0.56,1.51) | 0.83 (0.48,1.43) |  |  |  |
| 2021 vs 2019 | 1.24 (0.77,1.98) | 0.98 (0.61,1.57) | 0.94 (0.56,1.58) |  |  |  |
| 2020 vs 2019 | 1.17 (0.69,1.97) | 1.07 (0.63,1.80) | 1.13 (0.64,2.01) |  |  |  |

Models were adjusted for province of residence age, gender, ethnicity/race, education, income adequacy, and survey device used.
